# Population pharmacokinetics and dosing optimization of teicoplanin in renal transplant patients

**DOI:** 10.1128/aac.01568-24

**Published:** 2025-04-23

**Authors:** Yangang Zhou, Jiawei Peng, Ping Xu, Feng Wang, Jun Xi, Hedong Zhang, Shanbiao Hu, Han Yan, Liang Tan, Hualin Cai, Bikui Zhang, Gongbin Lan

**Affiliations:** 1 Department of Pharmacy, The Second Xiangya Hospital, Central South University 620666, Changsha, Hunan, China; 2 Institute of Clinical Pharmacy, Central South University 12570, Changsha, Hunan, China; 3 Department of Kidney Transplantation, The Second Xiangya Hospital, Central South University 612365, Changsha, Hunan, China; 4 Hunan Demeter Instruments Co., Ltd., Changsha, Hunan, China; 5 Clinical Research Center for Organ Transplantation in Hunan Province, Changsha, Hunan, China; Providence Portland Medical Center, Portland, Oregon, USA

**Keywords:** teicoplanin, population pharmacokinetics, renal transplant, dosing optimization, Monte Carlo simulation

## Abstract

The objectives of this study were to investigate the population pharmacokinetic (PK) characteristics of teicoplanin in renal transplant patients and to provide recommendations for optimal teicoplanin dosing regimens. A total of 99 renal transplant patients with 386 plasma samples were enrolled (306 in development and 80 in validation). A population PK analysis and simulations were performed to identify the optimal teicoplanin doses needed to provide an 80% probability of target attainment at 72 h and 168 h using both a trough concentration target of >15 µg/mL and the ratio of 24 h area under the concentration-time curve to the minimum inhibitory concentration >610.4. Teicoplanin was well described by a two-compartment PK model. The final model parameter estimates for clearance, central compartment volume of distribution, intercompartmental clearance, and peripheral compartment volume were 0.711 L/h, 11.3 L, 4.22 L/h, and 35.2 L, respectively. Creatinine clearance (CrCL) was the only covariate that significantly affected teicoplanin clearance. Dosing simulation results showed that standard dosing regimens were unable to meet the treatment needs of all patients, and CrCL-based individual dosing regimens are recommended for both loading dose and maintaining dose. Higher-than-standard teicoplanin doses are necessary to achieve prompt and appropriate drug exposure in renal transplant patients.

## INTRODUCTION

Gram-positive bacteria, especially methicillin-resistant *Staphylococcus aureus* (MRSA) infections, are common in renal transplant patients (1, 2). Vancomycin and teicoplanin are the glycopeptides currently recommended for the treatment of these infections (3–5). Comparative studies have revealed that there is no significant discrepancy in antimicrobial activity between teicoplanin and vancomycin, but fewer adverse effects such as nephrotoxicity (6, 7) and infusion reaction were observed with teicoplanin (8, 9). Given the increased susceptibility to drug-induced nephrotoxicity in renal transplant patients, teicoplanin represents a more promising therapeutic option for this population.

Teicoplanin strongly binds to plasma albumin, and it has an extremely long elimination half-life ranging from 83 to 163 h (10–12). Consistent with these pharmacokinetic (PK) characteristics, wide variations and fluctuations of concentrations are expected when administering fixed-dose regimens, and individualized doses are essential to achieve therapeutically effective concentration. Several reports have been published with the intent of defining the most appropriate regimen in different patient populations using model-informed precision dosing (MIPD) strategy, and wide variations of PK parameters were observed in different patients (13–18). Kang et al. (14) found slower teicoplanin clearance in elderly critically ill patients, while Wang and Wi et al. (15, 17) observed larger peripheral volume of distribution in patients with sepsis and receiving venoarterial extracorporeal membrane oxygenation (ECMO). Therefore, diverse teicoplanin dosing nomograms were established among different patients.

Different pharmacokinetic characteristics are anticipated in renal transplant patients depending on the unique physiologic status of these patients (18, 19). Moreover, in the early postoperative phase following kidney transplantation, patients undergo gradual recovery of renal function, accompanied by increased urine output and a rapid decline in serum creatinine levels, typically returning to normal within 2–4 weeks (20–22). These significant fluctuations in renal function further complicate the prediction of teicoplanin concentrations. In addition, renal transplant patients are more sensitive to adverse drug reactions (ADRs). For example, at equivalent dosages, renal transplant patients receiving polymyxin B demonstrate significantly higher incidences of neurological toxicity (63.4% vs. 27%) and pigment toxicity (51.5% vs. 15%) compared to other patients (23). Furthermore, within the recommended therapeutic target of voriconazole, ADRs such as hallucinations (63.64%), insomnia (55.84%), and visual impairment (44.16%) occur at markedly higher rates than in other patients (9%–16%) (24, 25). These findings suggest that the pharmacokinetic/toxicodynamic (PK/TD) profile in renal transplant patients differs significantly from that in non-transplant individuals. Therefore, the present recommended regimens might not be suitable for renal transplant patients, which might lead to decreased efficacy or a higher risk of toxicity, especially nephrotoxicity. To date, a comprehensive dosing regimen for teicoplanin tailored to the population PK of renal transplant patients remains unavailable. Our study aimed to investigate the PK characteristics of teicoplanin in this patient population, identify and quantify factors contributing to PK variability, and develop optimal dosing regimens to ensure prompt and appropriate drug exposure.

## MATERIALS AND METHODS

### Study design and patients

This prospective study was conducted at the kidney transplantation department in the Second Xiangya Hospital of Central South University (Changsha, China). Renal transplant patients (aged ≥18 years) who received intravenous teicoplanin (Zhejiang Medicine Co., Ltd, Zhejiang, China) for ≥72 h were enrolled. The exclusion criteria were as follows: (i) lack any of the demographic and laboratory data described below, (ii) unable to get the blood samples, and (iii) pregnancy. This study was approved by the Ethics Committee of the Second Xiangya Hospital, Central South University, and informed consent was obtained from all patients or legal representatives of the patients. The study employed a two-phase enrollment design to establish and validate the population PK model. Initial model development utilized patient data collected between January 2022 and December 2023. Following the establishment of the final population PK model, a distinct cohort of patients was prospectively recruited between November 2024 and March 2025 to conduct an external validation.

Demographic and clinical characteristics were collected, including age, sex, height, body weight, hemoglobin (HGB), alanine aminotransferase (ALT), aspartate aminotransferase (AST), total bilirubin (TBIL), direct bilirubin (DBIL), serum albumin concentrations (ALB), blood urea nitrogen (BUN), serum creatinine concentrations (Scr). Creatinine clearance (CrCL) was calculated by the Cockcroft-Gault formula. In addition, other clinical information including post-transplantation time, which is defined as days from the transplantation surgery to the initial of teicoplanin treatment, the continuous renal replacement therapy (CRRT) status, and daily urinary output during teicoplanin treatment were also recorded.

### Dosing regimen and blood sampling

Teicoplanin was given to all patients empirically as three loading doses of 400 mg every 12 hours, followed by a maintenance dose of 400 mg once daily for at least 3 days, and the infusion time was 1 h. For perioperative antimicrobial prophylaxis, teicoplanin administration was initiated 2 hours prior to the renal transplant procedure. Two to four blood samples (2 mL) were randomly collected immediately before the seventh dose of teicoplanin and at 0, 1, 3, 5, 7, 9, 11, 13, 14, 17, 18, 21, and 22 h after the end of infusion. The supernatant was immediately stored at −80℃ until analysis.

### Bioanalytical assay

The concentration of teicoplanin was determined using an automatic two-dimensional liquid chromatography-tandem mass spectrometry (2LC-MS/MS) method (Demeter Instrument Co., Ltd., Hunan, China). Teicoplanin concentrations were quantitated based on teicoplanin A2-2, which is the major isoform of teicoplanin components. Plasma samples (200 µL) were extracted by protein precipitation with MARG-1A (500 µL), after vortex oscillation for 1 minute, centrifugation at 14,500 r/min for 8 min, and 600 µL of the clear supernatant was transferred into autosampler vials for further analysis.

The standard solution of teicoplanin itself was employed as the internal standard (IS). Each time the sample was injected for analysis, the full-automatic coupling instrument of two-dimensional liquid chromatography extracts a set dose of sample solution as IS and stores it in the coupled instrument. Then, the stored IS will be injected into the chromatographic column 0.42 minutes later than sample injection via the valve switching device. Therefore, the sample and IS were differentiated by different peak time.

The two-dimensional separation conditions consisted of the following: the chromatographic column was a MSCB-2A column (3.0  ×  100 mm, 3.5 µm, ANAX, Changsha, China); the mobile phase was a 45:55 (vol/vol) solution of MMA-2D and MMB-2A at a flow rate of 0.6 mL/min. The column temperature was 45℃, and the injection volume was 1 µL. The concentration of teicoplanin in plasma was determined by the ESI ion source and positive ion mode multi-reaction monitoring method (MRM). The ion transitions were performed at m/z 940.4→316.1 for teicoplanin A2-2 with the collision voltage of 9 eV. The selected mass transitions were 500 m/z.

The method was validated in terms of its selectivity, accuracy, within- and between-run precision, recovery, linearity, sensitivity, and stability. The calibration curve equation was y = 0.0405 x +−0.00533 (r = 0.9998) in the range of 5.0–100 µg/mL, and the LLOD of teicoplanin was 0.5 µg/mL. The intra-day and inter-day coefficients of variation were ≤3.72%.

### Population pharmacokinetic modeling

The Phoenix NLME program (version 8.1., Pharsight, A Certara Company, USA) with the method of first-order conditional estimation-extended least square method (FOCE-ELS) was used to develop the population PK model by analyzing teicoplanin concentration. One-, two-, and three-compartment models with first-order elimination were evaluated as potential structural PK models based on changes in the objective function value (OFV, −2 × log-likelihood) and goodness-of-fit plots. The inter-individual variability of the PK parameters was evaluated using an exponential model. Residual variability was selected with additive, proportional, and combined error models:


$$\begin{array}{c} \ln\left(Y_{ij}\right)=\ln\left(F_{ij}\right)+\varepsilon_{2,ij}/F_{ij}\\ \ln\left(Y_{ij}\right)=\ln\left(F_{ij}\right)+\varepsilon_{1,ij}\\ \ln\left(Y_{ij}\right)=\ln\left(F_{ij}\right)+\varepsilon_{1,ij}+\varepsilon_{2,ij}/F_{ij} \end{array}$$

The following patient covariates were tested for their influence on the PK parameters of teicoplanin: age, sex, body weight, ALT, AST, TBIL, DBIL, ALB, baseline, and real-time CrCL. The stepwise covariate modeling (SCM) approach was used to test the covariate model in this analysis. A reduction in OFVs of ≥3.84 was considered to be statistically significant (*P* < 0.05) for the inclusion of one covariate in the forward inclusion steps, and an increase in OFVs of ≥6.63 was considered to be statistically significant (*P* < 0.01) in the backward elimination steps.

### Model evaluation and validation

The validity of the final model was assessed by visual inspection of goodness-of-fit (GoF) plots, including observed concentrations versus individual predictions (IPRED), observed concentrations versus population predictions (PRED), and conditional weighted residuals (CWRES) versus population predictions and time. Next, the accuracy and stability of the final model were evaluated using the bootstrap method. The medians and 95% confidence intervals of the PK parameter estimates were obtained from the 1,000 bootstrap runs. The model was considered stable if the typical population values for the PK parameters of the final model were within the 95% confidence interval of the bootstrap results, and the biases were less than 10% with no bias across zero. Furthermore, a prediction-corrected visual predictive check (pc-VPC) was performed with 1,000 data set simulations, after which the 5th to 95th percentiles of the simulated teicoplanin concentrations were overlaid with the observed data to assess the predictive performance of the final model.

To assess external validity, GoF plots and pc-VPC diagnostic assessments were performed as they were used for internal evaluation. In addition, bias and precision were calculated by computing prediction error (PE), mean prediction error (MPE), and root mean square error (RMSE) between the measured teicoplanin concentrations of the external data set and those predicted by the final model. The calculation equations were as follows:


$$\begin{array}{c} PE=PRED_{i}-Obs_{i}\\ MPE=\sum \frac{PRED_{i}-Obs_{i}}{N}\\ RMSE=\sqrt{\sum \frac{{\left(PRED_{i}-Obs_{i}\right)}^{2}}{N}} \end{array}$$

where PRED is individual predicted concentrations, Obs is observed concentrations, and *N* denotes the total number of observations.

### Monte Carlo simulations and dosing optimization

Monte Carlo simulations were performed using the parameter estimates from the final population PK model to create plasma teicoplanin concentration-time profiles. Four teicoplanin loading doses ranging from 400 to 1,000 mg, administered every 12 h for either three or five doses with maintenance doses ranging from 200 to 1,000 mg, administered per 24–48 h to included subjects were studied. Five renal function levels of CrCL ≤10, 10–30, 30–60, 60–90, and 90–120 mL/min were simulated, and at each CrCL level, 1,000 simulations were performed for each candidate regimen. Trough concentrations at 72 h (C_min,72 h_) and 168 h (C_min,168 h_) after the initial loading dose, as well as an area under the concentration-time curve from time 72 to 96 h (AUC_72–96 h_), and from time 168 to 192 h (AUC_168–192 h_) were calculated for each simulated dosing regimen. The probability of target attainment (PTA) was calculated for each dosing regimen using the two efficacy targets: (i) C_min_ ≥15 mg/L and (ii) AUC_24h_/MIC ≥ 610.4 (26); as well as the toxicity threshold of C_min_ ≥40 mg/L (26). The optimal dosing regimen was chosen to be the lowest dose required to maintain a PTA ≥ 80% for both efficacy targets and a PTA ≤ 10% for toxicity.

## RESULTS

### Patient characteristics

A total of 99 patients were enrolled in the present study, where 79 of them were grouped into the development data set and 20 patients were grouped into the external validation data set. The demographic information and baseline characteristics of the patients are shown in Table 1. The median age was 42.0, and hypertension (81.0%) and diabetes (25.3%) were the most common comorbidities. None of these patients were on mechanical ventilation and renal replacement therapy. The median teicoplanin treatment duration was 9 days, and 80 patients used it for perioperative prophylaxis while 19 patients for the treatment of confirmed or probable gram-positive bacterial infection (Table S1).

**TABLE 1:** Demographic information and baseline characteristics of all enrolled patients^*b*^

Variable	Development dataset^*a*^ (*n* = 79)	Validation dataset^*a*^ (*n* = 20)	Overall^*a*^ (*n* = 99)
Demographic			
Gender (male)	56 (70.9%)	16 (80.0%)	72 (72.7%)
Age (year)	42.5 (IQR: 36, 52.8)	42.3 (IQR: 35, 53)	42.0 (IQR: 36, 53)
Height (cm)	168 (IQR: 160, 173)	170 (IQR: 165, 172)	169 (IQR: 160, 173)
Weight (kg)	64.8 (IQR: 54, 74.8)	56.3 (IQR: 52.4, 67.5)	62.7 (IQR: 53.7, 72.0)
Clinical condition			
Time after transplantation (month)			
≤ 1	65	15	80
1–12	4	1	5
＞12	10	4	14
Concomitant immunosuppressive agents			
Tacrolimus	79 (100%)	20 (100%)	99 (100%)
Mycophenolate mofetil	79 (100%)	20 (100%)	99 (100%)
Baseline serum albumin concentration (g/L)	37 (IQR: 34.4, 39.5)	36.8 (IQR: 33.7, 38.2)	37 (IQR: 34.2, 39.1)
Baseline HGB (g/L)	105 (IQR: 92, 117)	106 (IQR: 94, 118)	105 (IQR: 92, 117.3)
Baseline creatinine clearance (mL/min)	9 (IQR: 6.6, 13.8)	6.6 (IQR: 5.6, 14.3)	6.8 (IQR: 6.1, 14.3)
Baseline BUN (mmol/L)	18.8 (IQR: 12.8, 26.1)	16.9 (IQR: 13.1, 27.7)	18.7 (IQR: 12.8, 26.6)
Baseline ALT (U/L)	12.9 (IQR: 8.7, 16.6)	10.9 (IQR: 6.8, 18.5)	12.8 (IQR: 7.6, 16.8)
Baseline AST (U/L)	14.2 (IQR: 9.2, 19.5)	14.7 (IQR: 11, 23.2)	14.4 (IQR: 10, 19.7)
Baseline TBIL (μmol/L)	6.3 (IQR: 4.9, 9)	6.8 (IQR: 4.8, 10.4)	6.5 (IQR: 4.9, 9.2)
Baseline DBIL (μmol/L)	2.4 (IQR: 1.8, 3.6)	2 (IQR: 1.6, 2.8)	2.3 (IQR: 1.7, 3.5)
Teicoplanin treatment duration (days)	9 (IQR: 8, 12)	2.3 (IQR: 2.1, 6.4)	9 (IQR: 7, 12)

^*a*^ Categorical data are number (%) of subjects, continuous data are expressed as median (interquartile range, IQR).

^*b*^ IQR, interquartile range; HGB: hemoglobin; BUN: blood urea nitrogen; ALT: alanine aminotransferase; AST: aspartate aminotransferase; TBIL: total bilirubin; DBIL: direct bilirubin.

### Population pharmacokinetic modeling

The population PK model was developed based on 306 plasma concentrations obtained from 79 patients, each patient on average contributed four clinical samples. The samples collected at different times are summarized in Fig. S1.

The PK profile of teicoplanin was well described by a two-compartment model with first-order elimination with proportional residual variability and interindividual variability. The PK parameters estimated included clearance from the central compartment (CL) and clearance between central compartment and peripheral compartment (CLd), the central (Vc), and peripheral (Vp) volume of distribution.

The impact of covariates on the Bayesian post hoc PK parameters from the final population PK basic model is provided in Table S2. Among the covariates tested, the inclusion of real-time CrCL in CL using a proportional model (change in objective function value [ΔOFV] 10.203) significantly improved the model fit. No other covariates were supported for inclusion in the final model. The final PK model equations were as follows:


$$\begin{array}{c} CL(L/h)=0.711\times (CrCL÷17{)}^{0.198}\times e^{\eta 1}\\ Vc(L)=11.3\times e^{\eta 2}\\ CLd(L/h)=4.22,Vp(L)=35.3 \end{array}$$

The typical values of CL, Vc, CLd, and Vp were 0.711 L/h, 11.3 L, 4.22 L/h, and 35.2 L, respectively.

These values generally agreed with the median estimates obtained by the bootstrap method (Table 2).

**TABLE 2:** Population PK parameter estimates in the final model and bootstrap^*a*^

Final model			Bootstrap method (*n* = 992)		
Parameters (Unite)	Estimate	Standard error (%SE)	Parameters (Unite)	Estimate (median)	90% CI
Fixed effects			Fixed effect		
CL (L/h)	0.711	6	CL (L/h)	0.714	0.614–0.802
CLd (L/h)	4.22	11	CLd (L/h)	4.22	3.36–5.24
Vc (L)	11.3	7	Vc (L)	11.3	9.93–12.8
Vp (L)	35.3	10	Vp (L)	35	28.4–42.8
CrCLCL	0.198	30	CrCLCL	0.196	0.073–0.319
Random effects (%)			Random effects (%)		
ω_CL_	40.1	12	ω_CL_	39.6	30.6–49.2
ω_Vc_	37.3	21	ω_Vc_	36.2	16.7–53.0
Residual error (%)			Residual error (%)		
Proportional residual (%)	14.6	14	Proportional residual (%)	14.3	10.4–18.2

^*a*^ CI: confidence interval; CL: the clearance in the central compartment; CLd: the clearance between the central compartment and peripheral compartment; Vc: the volume distribution in the central compartment; Vp: the volume in the peripheral compartment; CrCLCL: the influence coefficient of CrCL on CL; ω: Interindividual variation.

### Model evaluation and validation

**Fig 1:**
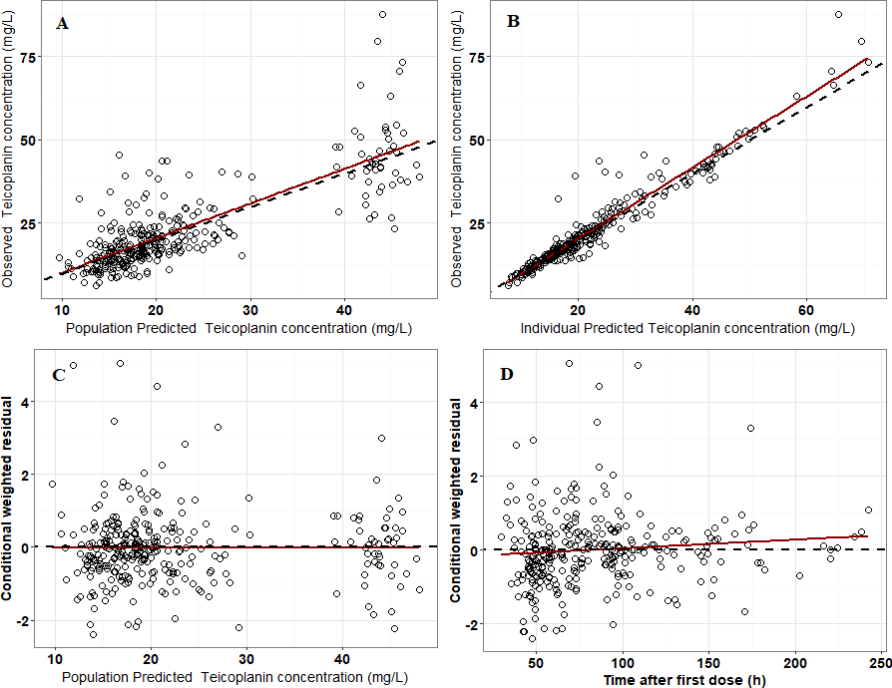
Goodness-of-fit plots for the final population pharmacokinetic model. (**A**) Observed versus population predicted concentrations (PRED); (**B**) Observed versus individual predicted concentrations (IPRED); (**C**) Conditional weighted residuals (CWRES) versus population predicted concentrations (PRED); (**D**) Conditional weighted residuals (CWRES) versus time after dose. The dashed line in the upper panel is y = x; the dashed line in the lower panel is y = 0. The solid red line is a linear regression line.

**Fig 2:**
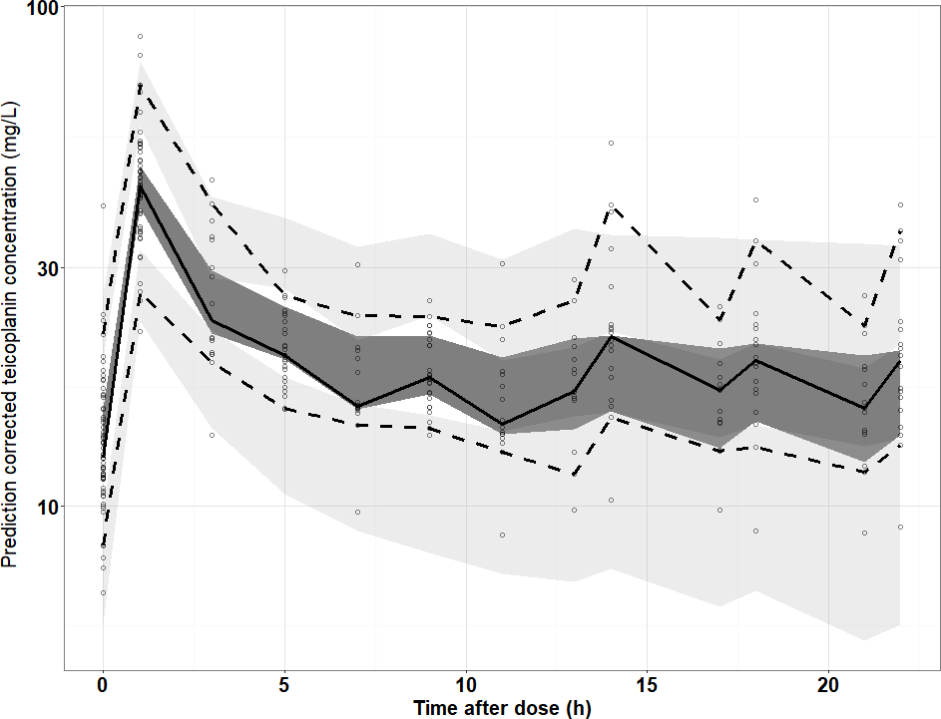
Pc-VPC results of the final population PK model. Open circles represent the observed concentrations, and the solid line, lower and upper dashed lines represent 50%, 5%, and 95% of the observed and predicted concentrations, respectively. The shaded area represents the simulation-based 90% confidence intervals.

The goodness-of-fit (GoF) plots in the final model are shown in Fig. 1. The plots showed that the structure of the final model was not biased and that the model was acceptable. In addition, most conditional weighted residual values were evenly distributed randomly around the line of unity (±6 standard deviations of the mean), which indicated the suitability of the error model. The results of pc-VPC showed that the 5th to 95th percentiles of the simulated data overlaid most of the observed data, supporting the predictive performance of the model (Fig. 2).

**Fig 3:**
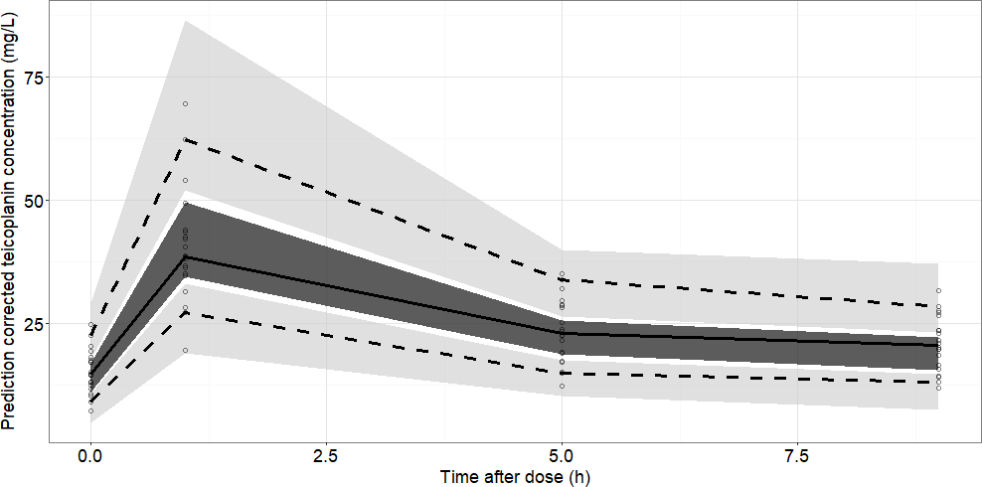
Pc-VPC results of the final model (validation data set). Open circles represent the observed concentrations, and the solid line, lower and upper dashed lines represent 50%, 5%, and 95% of the observed and predicted concentrations, respectively. The shaded area represents the simulation-based 90% confidence intervals.

External validation was performed with a data set of 80 samples from 20 patients, and the validation results demonstrated prediction accuracy with an RMSE of 8.49% and an MPE of 0.704%. GoF plots showed a decent agreement between observations with both population and individual predictions (Fig. S2A and B). No obvious trend was noted in the conditional weighted residuals (Fig. S2C and D). Pc-VPC of the final model indicated that external values were contained within the 95% prediction intervals, suggesting the final model had adequate predictive ability over the validation data set (Fig. 3). All these results suggested that the final model exhibited adequate predictive performance.

### Monte Carlo simulations

Table 3 shows the PTAs at the target of efficacy and toxicity at 72 h and 168 h after the initial teicoplanin treatment using different dosing regimens, stratified by CrCL. Overall, a trend toward an increase in the PTA with increasing doses and decreasing renal function was noted. As expected, the results of loading dosage regimens were mainly reflected in C_min,72h_, while the results of the maintenance dosage regimens were mainly reflected in C_min,168h_. To balance the efficacy and toxicity, for patients with CrCL <10 mL/min, the recommended dosing regimen was 400 mg q12h for five times or 600 mg q12h for three times, followed by 400 mg q24h. For patients with CrCL 10–30 mL/min, the recommended dosing regimen was 600 mg q12h for three times, followed by 600 mg q24h. For patients with CrCL 30–60 mL/min, the recommended dosing regimen was 800 or 1,000 mg q12h for three times, followed by 600 mg q24h. For patients with CrCL 60–90 mL/min, the recommended dosing regimen was 800 mg q12h for three times, followed by 800 mg q24h. For patients with CrCL 90–120 mL/min, the recommended dosing regimen was 800 mg q12h for five times or 1,000 mg q12h for three times, followed by 800 mg q24h.

**TABLE 3:** Monte Carlo simulation results stratified by CrCL^*a*^

CrCL (ml/min)	Dose regimen	PTA (%) of C_min,72 h_＞15 µg/mL	PTA (%) of C_min,168 h_＞15 µg/mL	PTA (%) of C_min,72 h_＞40 µg/mL	PTA (%) of C_min,168 h_＞40 µg/mL
<10	400 mg q12h × 5, 200 mg QD	91.8	56.7	0	0
	400 mg q12h × 3, 400 mg QD	70.7	83.9	0	1.6
	**400 mg q12h × 5, 400 mg QD**	**91.8**	**86.8**	**0**	**4.8**
	600 mg q12h × 3, 200 mg QD	84.1	50.5	0	0
	600 mg q12h × 5, 200 mg QD	99.3	71.9	18.5	2.4
	**600 mg q12h × 3, 400 mg QD**	**92.2**	**87.1**	**0**	**5.5**
	600 mg q12h × 5, 400 mg QD	99.3	90.2	18.5	15.2
	600 mg q12h × 3, 600 mg QD	96.4	97.2	0.6	28.5
10–30	600 mg q12h × 3, 400 mg QD	76.5	66.5	0	0.8
	600 mg q12h × 5, 400 mg QD	96.2	75.3	5.2	4.0
	**600 mg q12h × 3, 600 mg QD**	**86.8**	**89.0**	**0**	**9.4**
	600 mg q12h × 5, 600 mg QD	95.8	89.8	5.2	15.2
	800 mg q12h × 5, 200 mg QD	99	57.6	40.1	1.9
	800 mg q12h × 3, 400 mg QD	89.2	71.6	1.0	2.0
	800 mg q12h × 5, 400 mg QD	99.0	79.6	40.1	9.2
	800 mg q12h × 3, 600 mg QD	92.9	88.8	3.0	13.2
30–60	600 mg q12h × 3, 600 mg QD	73.5	76.6	0	3.2
	600 mg q12h × 5, 600 mg QD	89.4	78.9	1.7	6.6
	800 mg q12h × 5, 400 mg QD	97.9	65.4	23.1	3.5
	**800 mg q12h × 3, 600 mg QD**	**84.8**	**77.4**	**0.7**	**4.7**
	800 mg q12h × 5, 600 mg QD	96.8	80.1	22.5	11.7
	800 mg q12h × 3, 800 mg QD	90.6	90.0	2.3	18.0
	**1,000 mg q12h × 3, 600 mg QD**	**91.7**	**80.4**	**6.7**	**8.0**
	1,000 mg q12h × 3, 800 mg QD	94.6	89.9	11.3	20.4
60–90	600 mg q12h × 3, 600 mg QD	64.3	67.8	0.0	2.3
	600 mg q12h × 5, 600 mg QD	86.8	72.4	0.9	4.1
	800 mg q12h × 5, 600 mg QD	94.7	73.2	15.1	7.2
	**800 mg q12h × 3, 800 mg QD**	**82.9**	**82.3**	**1.3**	**10.7**
	800 mg q12h × 5, 800 mg QD	94.9	84.5	16.6	17.7
	1,000 mg q12h × 3, 600 mg QD	86.2	70.3	4.1	5.0
	1,000 mg q12h × 3, 800 mg QD	90.5	85.0	6.3	12.7
	1,000 mg q12h × 3, 1000 mg QD	93.7	92.6	12.7	27.9
90–120	800 mg q12h × 5, 600 mg QD	91.6	64.6	10.0	3.8
	800 mg q12h × 3, 800 mg QD	79.6	79.0	0.7	7.6
	**800 mg q12h × 5, 800 mg QD**	**91.8**	**79.8**	**9.5**	**10.2**
	1,000 mg q12h × 3, 600 mg QD	81.9	61.8	2.0	2.5
	**1,000 mg q12h × 3, 800 mg QD**	**85.7**	**79.2**	**4.5**	**9.7**
	1,000 mg q12h × 5, 800 mg QD	97.6	83.2	31.2	13.8
	1,000 mg q12h × 3, 1000 mg QD	90.3	88.8	9.2	21.2
	1,000 mg q12h × 5, 1000 mg QD	97.1	89.0	32.4	24.6

^*a*^ The bold fonts indicate dosage regimens that both meet the 15 µg/mL ＜ C_min_ ＜40 µg/mL standard.

**Fig 4:**
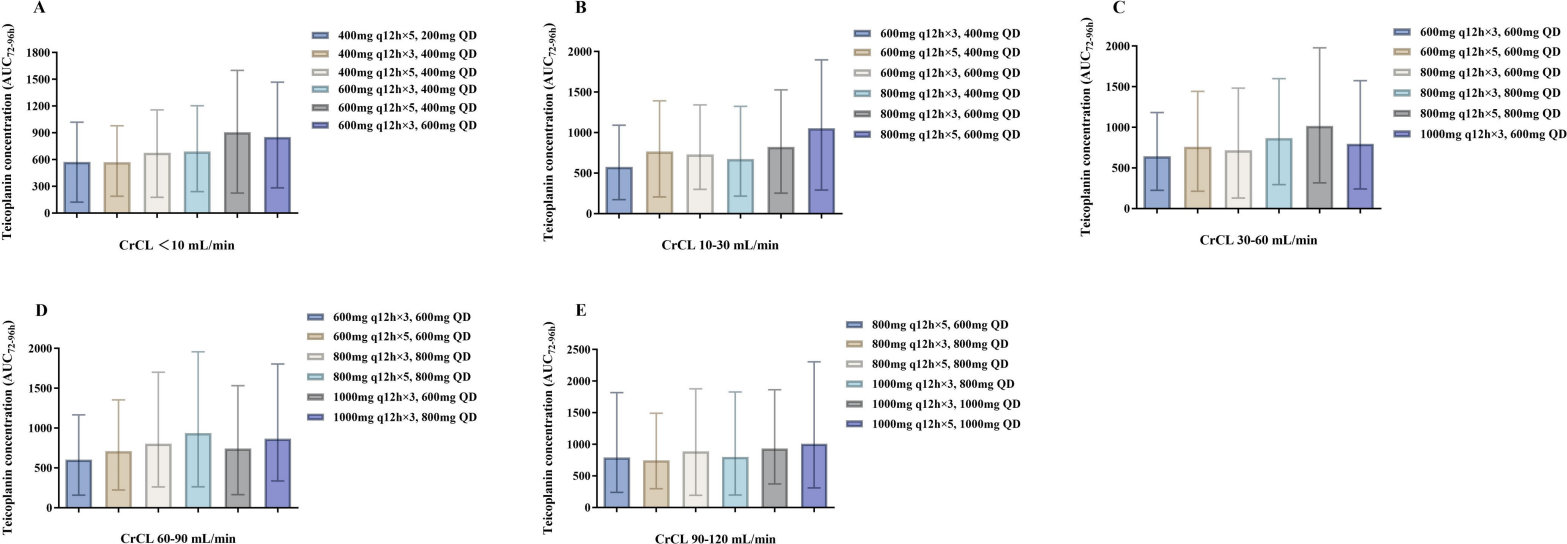
Teicoplanin AUC_72–96 h_ with different dose regimens in subgroups stratified by CrCL. (**A-E**) represent AUC_72–96 h_ in subgroups with CrCL ≤10, 10–30, 30–60, 60–90, and 90–120 mL/min, respectively. Each bar represents the median and range of AUC_24 h_.

**Fig 5:**
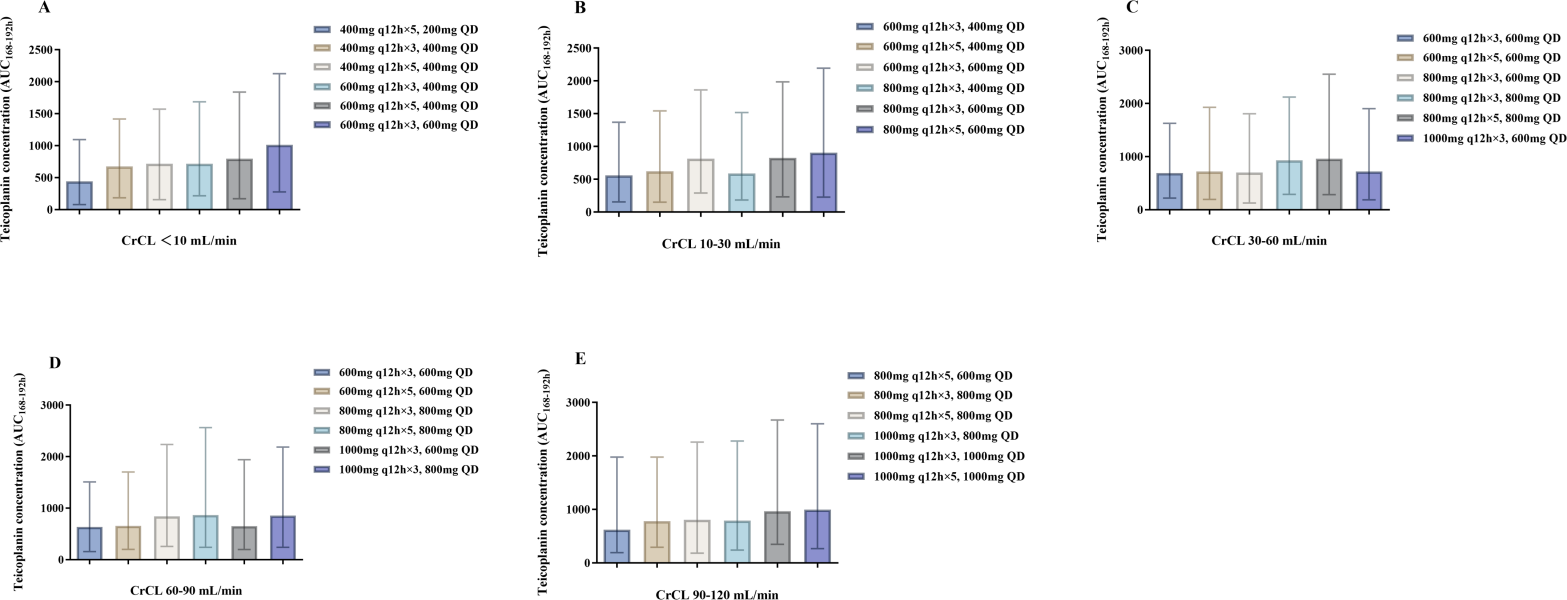
Teicoplanin AUC_168-192h_ with different dose regimens in subgroups stratified by CrCL. (**A–E**) represent AUC_168–192 h_ in subgroups with CrCL ≤10, 10–30, 30–60, 60–90, and 90–120 mL/min, respectively. Each bar represents the median and range of AUC_24 h_.

**Fig 6:**
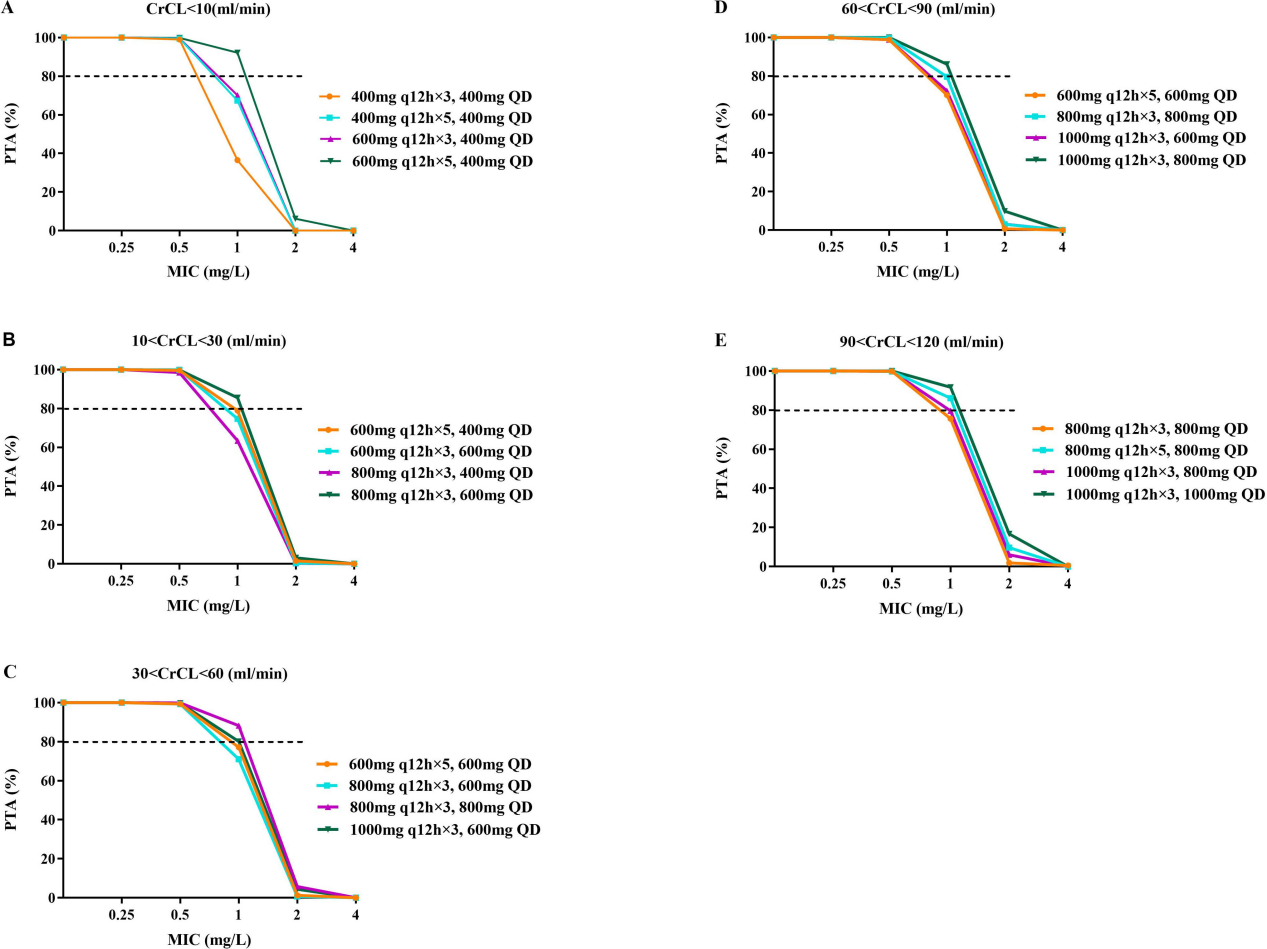
PTA of teicoplanin for the target of AUC_72-96h_/MIC ≥610.4 in subgroups stratified by GFR. (**A–E**) represent subgroups with CrCL ≤10, 10–30, 30–60, 60–90, and 90–120 mL/min, respectively. The dashed black line indicates the target PTA of 80%.

**Fig 7:**
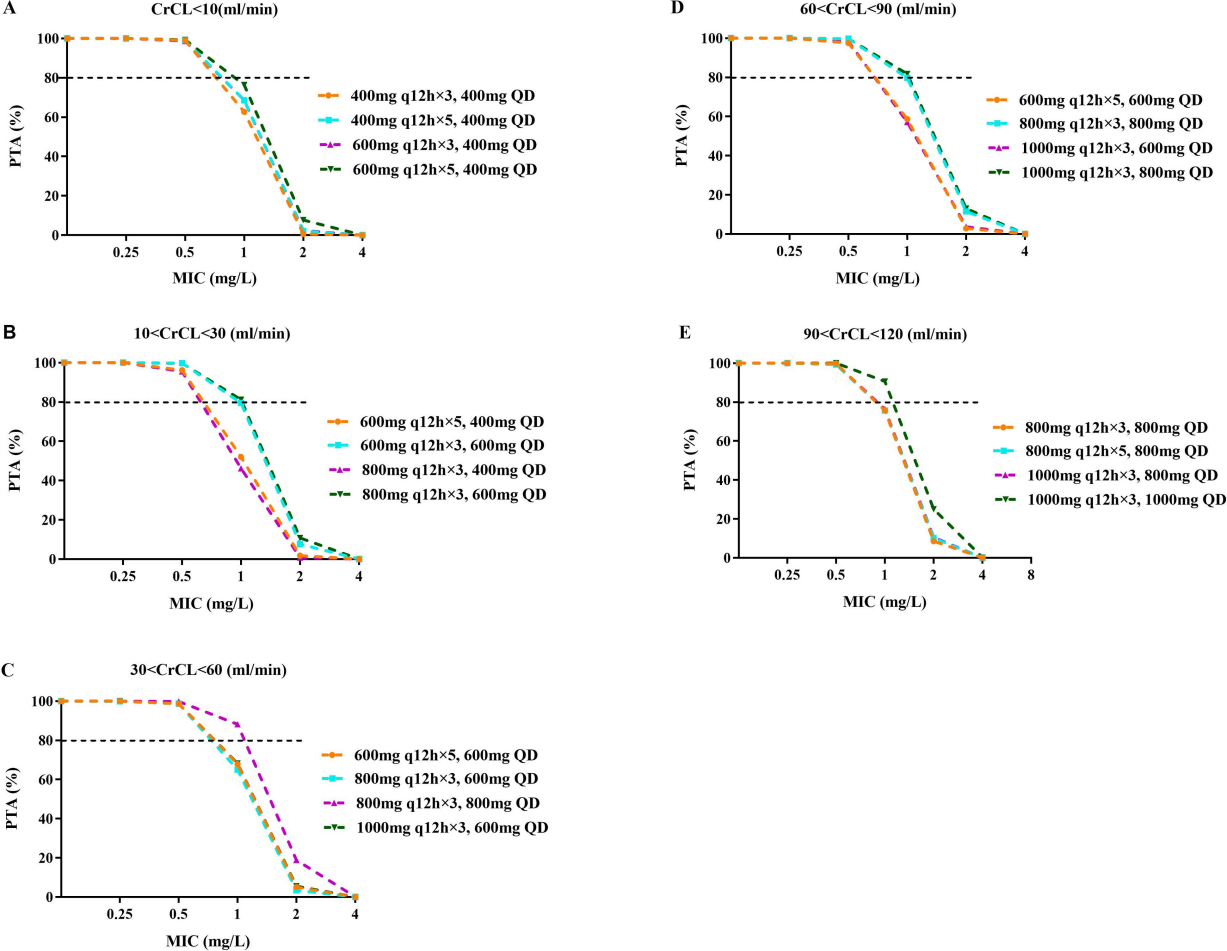
PTA of teicoplanin for the target of AUC_168-192h_/MIC ≥ 610.4 in subgroups stratified by GFR. (**A–E**) represent subgroups with CrCL ≤10, 10–30, 30–60, 60–90, and 90–120 mL/min, respectively. The dashed black line indicates the target PTA of 80%.

Figures 4 and 5 depict the median AUC_72–96 h_ and AUC_168–192 h_ achieved with different regimens in subgroups stratified by renal function, respectively. A dose-dependent increase in teicoplanin exposure was observed in all subgroups, with significant variation at the same dose. Figures 6 and 7 demonstrate teicoplanin PTAs at the target of AUC_24h_/MIC ≥610.4 in the time frame of 72–96 h and 168–192 h, respectively. In consistent with Table 3, loading and maintenance regimens were mainly associated with the exposure in the time frame of 72–96 h and 168–192 h, respectively. Moreover, since most of the regimens contained three times the loading dose, the maintenance dose regimen also contributed to the PTAs in the initial 3 days. When considering a target of PTA ≥ 80%, all of the selected regimens achieved the PTA target when MIC = 0.5 mg/L. However, when MIC = 1.0 mg/L, higher dosages were needed to achieve the PTA target. For patients with CrCL <10 mL/min, the recommended dosing regimen was 600 mg q12h for five times, followed by 400 mg q24h. For patients with CrCL 10–30 mL/min, the recommended dosing regimen was 800 mg q12h for three times, followed by 600 mg q24h. For patients with CrCL 30–60 mL/min, the recommended dosing regimen was 800 mg q12h for three times, followed by 800 mg q24h. For patients with CrCL 60–90 mL/min, the recommended dosing regimen was 1,000 mg q12h for three times, followed by 800 mg q24h. For patients with CrCL 90–120 mL/min, the recommended dosing regimen was 1,000 mg q12h for three times, followed by 1,000 mg q24h. None of the simulated regimens achieved adequate target attainment at MICs at or above the EUCAST breakpoint of 2 mg/L.

## DISCUSSION

To our knowledge, this is the first prospective population PK study of teicoplanin in renal transplant patients. A two-compartment model with first-order elimination reasonably fitted the concentration-time data for teicoplanin, and Monte Carlo simulation provided the optimized regimens for teicoplanin in renal transplant patients with different renal functions.

It is known that the elimination of teicoplanin is triexponential, with extremely long half-lives of 83 to 163 h (10). However, due to the short sampling duration, our population PK model, as well as most of the others, could not depict the terminal elimination phase; thus, the PK of teicoplanin was best described by the two-compartment model with faster clearance and shorter half-life. These results are consistent with the feature of its rapid elimination in α and β phases (10, 11). In our study, the typical value of CL is within the range of previously reported values, but lower than in critically ill patients (0.723 VS 0.838–1.03 L/h). It mainly accounted for the severe renal impairment in our patients; besides, continuous renal replacement therapy (CRRT) was widely used in other studies, which could also accelerate the clearance of teicoplanin (27–29). The distribution in the present model is smaller than previous models (46.6 L VS 60.5–121.1 L) (14, 15, 17), it might be because most other models were comprised of critically ill patients, and factors such as endothelial damage and increased capillary permeability, compounded by a large amount of fluid in these patients, might increase the distribution volume.

Similar to the previous study, covariate analysis in this study demonstrated a significant positive association between teicoplanin CL and CrCL, and it is consistent with the fact that teicoplanin is mainly eliminated unchanged by the kidney. Several studies revealed weight as a significant covariate influencing teicoplanin elimination and distribution (30–32). However, we found no correlation between body weight and teicoplanin PK parameters in this study, which might be due to the limited samples of the population PK model and a relatively narrow distribution of patients’ weights (IQR 54–74.8 kg). Accordingly, Monte Carlo simulations were performed using fixed doses. Due to the high plasma protein binding rate, previous studies of teicoplanin population PK models revealed that the ALB level was a significant covariate on the distribution of the volume of teicoplanin, especially in critically ill patients and those with hematological malignancy (30, 33, 34). However, in the present study, patients’ ALB levels were within the normal range (37.0 g/L), and it was not difficult to speculate no influence of ALB on the PK of teicoplanin. Future population PK research with rich sampling schedules is needed to better illuminate the pharmacokinetic characteristics of teicoplanin.

Considering the high plasma protein binding rate (about 90%) and extremely long elimination half-life of teicoplanin, a loading dose for 2–3 days is mandatory for all patients on teicoplanin to achieve the optimal concentration more rapidly. After that, a suitable maintaining dose should be employed to sustain the target concentration. Traditionally, it has been reported that teicoplanin trough concentration of ≥10 mg/L is needed to be achieved for the successful treatment of MRSA infections, thus lower doses were recommended before (35–37). However, more and more studies have revealed that higher concentration is needed for a better clinical response rate, and increased doses are required accordingly (38–40). In consistent with these results, our Monte Carlo simulation showed that 800–1,000 mg q12h for three times, followed by 800 mg q24h, is needed in patients with normal renal function to achieve the target concentration.

At present, estimated glomerular filtration rate (eGFR)-based maintenance dosing regimen is recommended for teicoplanin; however, whether loading doses should be adjusted according to renal function is still controversial. For most drugs with long half-lives, a single loading dose is generally sufficient to rapidly attain therapeutic concentrations. As the loading dose is basically determined by the volume of distribution (Vd) and target concentration, no reduction of it in renal impairment patients is recommended (41). However, because of the longer administration of the loading dose than that of other antibiotics, the impact of renal function on teicoplanin clearance should be considered for the loading dose regimen. As demonstrated by our Monte Carlo simulations, adjusting both the loading and maintenance doses based on renal function is critical to achieving optimal therapeutic exposure while mitigating toxicity risks (Table 3), and it is in accordance with previous studies (30, 42, 43). Nevertheless, Nakano et al. and Chen et al. (13, 44) found that at least 800 mg (about 12 mg/kg) is required to achieve the target of C_min,72h_ ≥ 15 mg/L in critically ill patients regardless of renal function. This inconsistency might be due to the different PK characteristics among diverse populations, and further research is warranted to clarify the optimal dosing strategy.

In line with other models, our study observed significant inter-individual variabilities (IIV) in the PK parameters (ω_CL_: 40.1%, ω_Vc_: 37.3%), resulting in substantial variations and fluctuations of concentrations, even among patients with comparable renal function (Fig. 4 and 5). The importance of noting is that higher doses may be advantageous in achieving the desired concentration, but they can also elevate the risk of surpassing the toxicity threshold in patients with greater exposure. For example, the regimens provided to achieve a 90% PTA with C_min_ ≥ 15 mg/L may result in trough concentrations exceeding 40 mg/L in a significant proportion of patients (15%–40%) (Table 3). Therefore, to achieve a balance between efficacy and toxicity, we recommend using the lowest doses necessary to maintain an 80% PTA. This may explain why other studies have also chosen lower PTAs (50%–80%) as their targets (13–16). These findings emphasize the importance of considering the significant variability in teicoplanin exposure within the population when utilizing the MIPD tool for predicting optimal treatment regimens.

As a time-dependent antibacterial drug with a long post-antibiotic effect (PAE), the PK/PD index that best correlates with teicoplanin antibacterial activity is AUC_24h_/MIC, but considering that it is difficult to obtain multiple serum concentrations to determine the AUC_24 h_ in clinical settings, C_min_ is recommended as a surrogate marker for optimal treatment outcomes (26). Therefore, in this study, we investigated the PTAs for both C_min_ ≥ 15 mg/L and AUC_24_/MIC ≥610.4. The results showed that C_min_ ≥ 15 mg/L is a potential candidate for predicting AUC_24_/MIC ≥610.4 at MIC values ≤ 0.5 mg/L. However, when MIC = 1 mg/L, regimens provided an 80% PTA with C_min_ ≥ 15 mg/L is not enough to achieve AUC_24h_/MIC ≥610.4, higher doses are needed to attain the adequate antibacterial effects. This is in accordance with vancomycin that C_min_ ≥ 15 mg/L equals AUC_24h_/MIC ≥345 under the circumstance of MIC ≤1 mg/L (45, 46). These results demonstrate that to avoid inadequate systemic exposures and suboptimal antibacterial activity, it is essential to take bacterial susceptibility into account when using C_min_ as the alternative efficacy predictor.

Last but not least, although AUC_24_/MIC is known as the key PK/PD parameter for teicoplanin, the PK/PD target for efficacy remains controversial. Matsumoto et al. (47) reported that the treatment success rate for teicoplanin was 87% in patients achieving AUC_24h_/MIC ≥900. Ramos-Martín et al. (48) demonstrated that an AUC_24h_/MIC value of 610.4 for an MRSA strain with a MIC value of 0.5 mg/L was needed for bactericidal efficacy, while Bryne et al. (31) recommended an AUC_24h_ value of 700–800 mg·h/L. These inconsistent results are reasonable since it is well known that the PK/PD indices and targets of antibiotics are diverse among different types of infections. Therefore, we presented the median and variability of the AUC_24_ simulation results achieved with the various regimens in our results (Fig. 4 and 5), which make it possible to select the optimal regimen in accordance with the required target. Further investigation is needed to better evaluate the PK/PD relationship of teicoplanin.

This study has several limitations. First, due to the short sampling duration, our population PK model could not depict the terminal elimination phase. Second, the sample size was limited, which restricted the ability to evaluate the impact of covariates on the population PK parameters. Therefore, these data might not have been sufficient to provide robust PK parameter estimates for the overall renal patient population. Future studies are needed to validate the current findings and to evaluate the clinical efficacy and safety of the recommended dosing regimen. Third, we did not assess the free concentration of teicoplanin, which is considered to be pharmacologically active. Considering the high protein binding ratio, the unbound teicoplanin concentration is prone to vary largely with serum albumin concentrations and might not be in accordance with the total concentration. To better optimize teicoplanin dosage, further study illustrating PK characteristics of the free drug concentration is needed.

### Conclusions

In conclusion, this study developed a reliable population PK model for teicoplanin in renal transplant patients, and a model-based individual dosing regimen was proposed. Teicoplanin clearance is significantly associated with renal function, CrCL-based individual dosing regimens are recommended for both loading dose and maintaining dose. To achieve the balance of efficacy and toxicity, it is of great importance to consider the substantial variability in teicoplanin exposure within the population when designing the optimal treatment regimens.

## Data Availability

The data sets generated during and/or analyzed during the current study are available from the corresponding author upon reasonable request.

## AFTER EPUB

*[This article was published on 23 April 2025 with errors in the funding information. The funding was corrected in the current version, posted on 15 May 2025.]*

## SUPPLEMENTAL MATERIAL

Supplemental material aac.01568-24-s0001.docx Table S1 and S2, Fig. S1 and S2.

ASM does not own the copyrights to Supplemental Material that may be linked to, or accessed through, an article. The authors have granted ASM a non-exclusive, world-wide license to publish the Supplemental Material files. Please contact the corresponding author directly for reuse.
