# Supplementary material for: Population pharmacokinetics and dosing optimization of teicoplanin in renal transplant patients
Source: Antimicrob Agents Chemother. 2025 Apr 23;69(6):e01568-24. doi: 10.1128/aac.01568-24 (PMC12135518; doi:10.1128/aac.01568-24)
Supplement: Supplemental material — Table S1 and S2, Fig. S1 and S2. [file aac.01568-24-s0001.docx]

Table S1 Demographic data for 19 patients with confirmed or susceptible gram-positive bacterial infection

| Variable | Values^a^ |
| --- | --- |
| Type of infections |  |
| Pneumonia | 14 (73.7%) |
| Urinary tract infection | 5 (26.3%) |
| Comorbidities |  |
| Hypertension | 16 84.2%) |
| Diabetes mellitus | 7 (36.8%) |
| Chronic renal dysfunction | 5 (26.3%) |
| Pathogens (n=14) |  |
| Methicillin-resistant Staphylococcus aureus  Enterococcus faecium | 9 (64.3%)  5 (35.7%) |
| Teicoplanin MIC values (n=9)  Methicillin-resistant Staphylococcus aureus  Enterococcus faecium | 0.5-1  1 |

Abbreviations: MIC, minimum inhibitory concentration.

^a^ Categorical data is number (%) of subjects.

Table S2 Model building steps of teicoplanin population pharmacokinetic model in the renal transplant patients.

| Models | OFV | ΔOFV |
| --- | --- | --- |
| Two-compartment model with a zero-order input rate  (Base model) | 1246.3 | -- |
| CL-sex | 1245.7 | -0.6 |
| Vc-sex | 1245.6 | -0.7 |
| CL-DFLAG | 1246.3 | 0 |
| Vc-DFLAG | 1246.3 | 0 |
| CL-age | 1243.1 | -3.2 |
| Vc-age | 1243.8 | -2.5 |
| Vc-body weight | 1242.6 | -3.7 |
| CL-body weight | 1243.8 | -2.5 |
| CL-BTBIL^a^ | 1244 | -2.3 |
| Vc-BTBIL | 1245 | -1.3 |
| CL-BALB | 1245.7 | -0.6 |
| Vc-BALB | 1245 | -1.3 |
| CL-BALT^b^ | 1243 | -3.3 |
| Vc-BALT | 1245.8 | -0.5 |
| CL-BCRCL | 1240.8 | -5.5 |
| Vc-BCRCL | 1245.8 | -0.5 |
| CL-CRCL | 1236.1 | -10.2 |
| Vc-CRCL | 1246.2 | -0.1 |

Abbreviations: OFV, objective function value; CL, systemic clearance; Vc, volume of the central compartment; DFLAG, categorical covariate distinguishing postoperative duration intervals, dichotomized into two temporal strata: the short-term postoperative phase (≤1 month) and extended postoperative period (>1 month); BTBIL, baseline total bilirubin; baseline BALB, baseline albumin; BALT, baseline alanine aminotransferase; BCRCL, baseline creatinine clearance; CRCL, real-time creatinine clearance;

^a^, A strong collinearity was observed between baseline total bilirubin (BTBIL) and baseline direct bilirubin (DBIL), with correlation coefficient reaching 0.8 (>0.6), only BTBIL was retained for subsequent covariate analyses.

^b^, A strong collinearity was observed between baseline alanine aminotransferase (BALT) and baseline aspartate aminotransferase (BAST), with correlation coefficient reaching 0.8 (>0.6), only BALT was retained for subsequent covariate analyses.


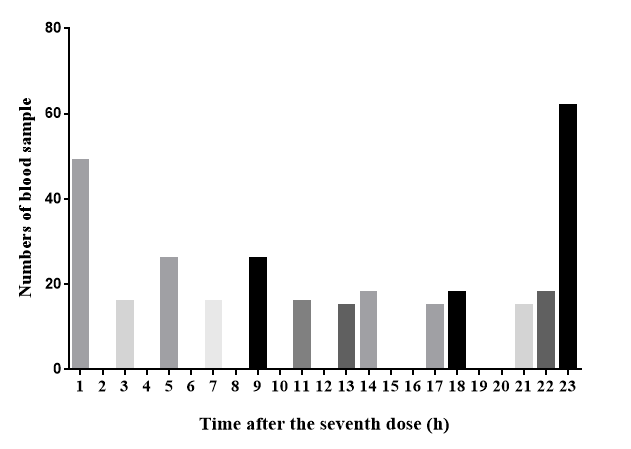


Figure S1 Time distribution of the serum samples. Each bar represents the samples collected at different times.


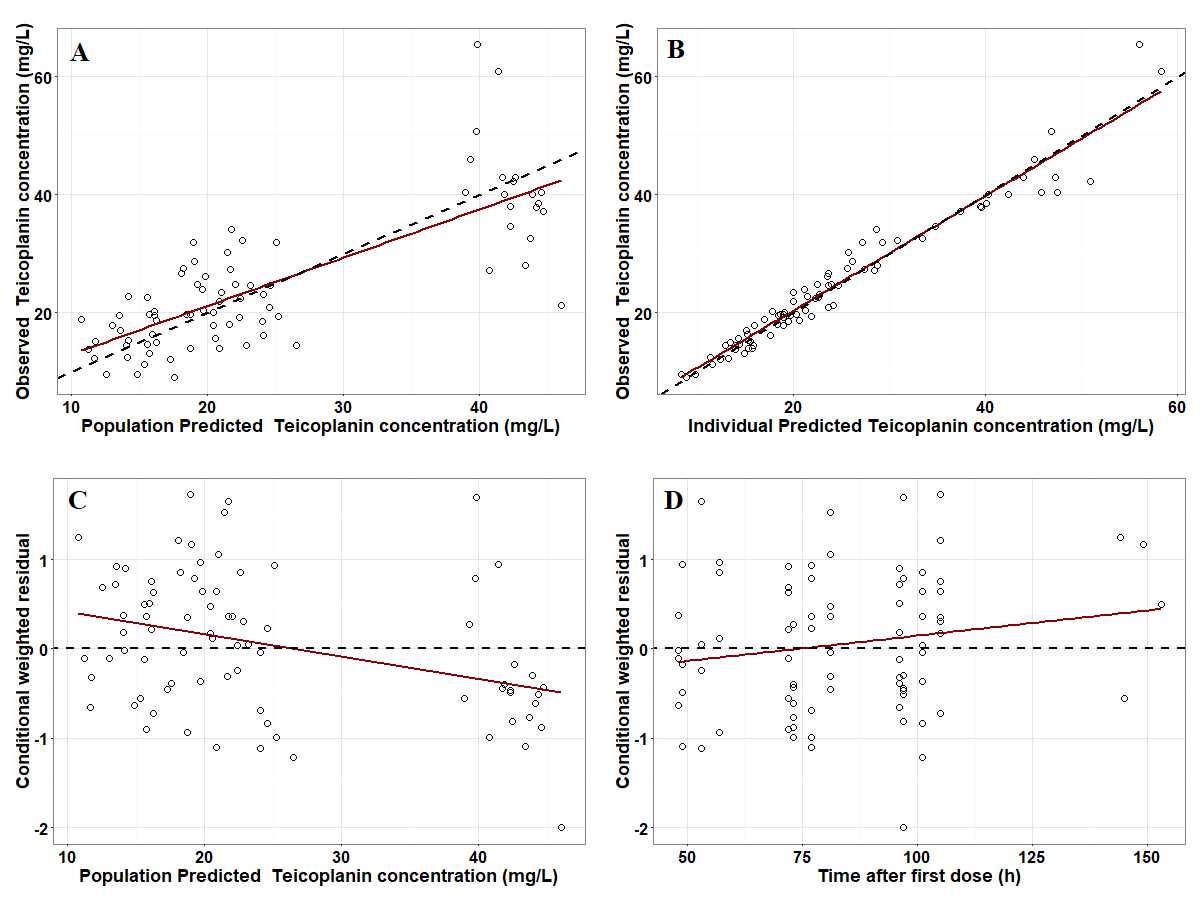


Figure S2 Goodness-of-ft plots of the final model (validation dataset). (A) Observed versus population predicted concentrations (PRED); (B) Observed versus individual predicted concentrations (IPRED); (C) Conditional weighted residuals (CWRES) versus population predicted concentrations (PRED); (D) Conditional weighted residuals (CWRES) versus time after dose. The dashed line in the upper panel is y=x; the dashed line in the lower panel is y=0. The solid red line is a linear regression line.
